# Serial prenatal MRI evaluation of valacyclovir-treated vs untreated fetuses with congenital cytomegalovirus (cCMV) infection

**DOI:** 10.1007/s00330-026-12698-y

**Published:** 2026-06-20

**Authors:** Mario Tortora, Elisa Fabbri, Alessia Arossa, Milena Furione, Luisa Chiapparini, Guglielmo Conti, Giana Izzo, Cecilia Parazzini, Filippo Arrigoni, Stefania Criscuolo, Fabio Tortora, Mariano Lanna, Andrea Righini, Chiara Doneda

**Affiliations:** 1 Neuroradiology Unit, Department of Advanced Biomedical Sciences, University of Naples “Federico II”, Naples, Italy; 2 Children’s Hospital V. Buzzi—ASST Fatebenefratelli-Sacco, Obstetrics and Gynecology Unit, Milan, Italy; 3 Department of Obstetrics and Gynecology, Fondazione IRCCS Policlinico San Matteo, Pavia, Italy; 4 Microbiology and Virology Unit, Fondazione IRCCS Policlinico San Matteo, Pavia, Italy; 5 Radiodiagnostic and Neuroradiology Unit, Fondazione IRCCS Policlinico San Matteo, Pavia, Italy; 6 Postgraduate School of Diagnostic and Interventional Radiology, Università Statale di Milano, Milan, Italy; 7 Paediatric Radiology and Neuroradiology Unit, Children’s Hospital V. Buzzi—ASST Fatebenefratelli-Sacco, Milan, Italy

**Keywords:** Congenital cytomegalovirus, Fetal MRI, Valacyclovir, Prenatal imaging, Temporal pole lesions

## Abstract

**Objectives:**

To compare serial prenatal MRI findings in valacyclovir-treated vs untreated fetuses with confirmed congenital cytomegalovirus (cCMV) infection, focusing on the occurrence and timing of minor brain lesions.

**Materials and methods:**

In this retrospective single-center cohort study, fetuses with confirmed cCMV infection and at least two prenatal brain MRI examinations were included. Fetuses with major brain malformations at baseline were excluded. Treated fetuses received oral valacyclovir (8 g/day) from diagnosis to delivery. Two senior neuroradiologists assessed MRI examinations in consensus, focusing on minor lesions (temporal pole T2 hyperintensity, focal temporal horn dilation, small cysts). Lesion timing was categorized as absent, late-onset, or early-onset. Statistical analyses included Fisher’s exact test, χ²-test, and odds ratios.

**Results:**

Seventy-seven fetuses were included (62 untreated, 15 treated). Persistently negative MRI findings were observed in 73.3% of treated fetuses vs 37.1% of untreated fetuses (*p* = 0.018; OR 4.67, 95% CI: 1.29–16.9). Early-onset minor lesions were observed only in untreated fetuses (11.3%). A significant difference between groups was confirmed when analysis was restricted to temporal pole lesions (*p* = 0.02).

**Conclusion:**

Maternal valacyclovir therapy was associated with a significantly higher likelihood of persistently normal prenatal MRI findings and absence of early minor brain lesions, suggesting a protective effect on fetal brain involvement in cCMV.

**Key Points:**

***Question*** *Minor temporal pole lesions are common early MRI findings in cCMV*.

***Findings*** *Valacyclovir-treated fetuses showed fewer and later-onset minor lesions*.

***Clinical relevance*** *Serial fetal MRI may serve as an imaging surrogate marker of treatment response*.

**Graphical Abstract:**

**Figure:**
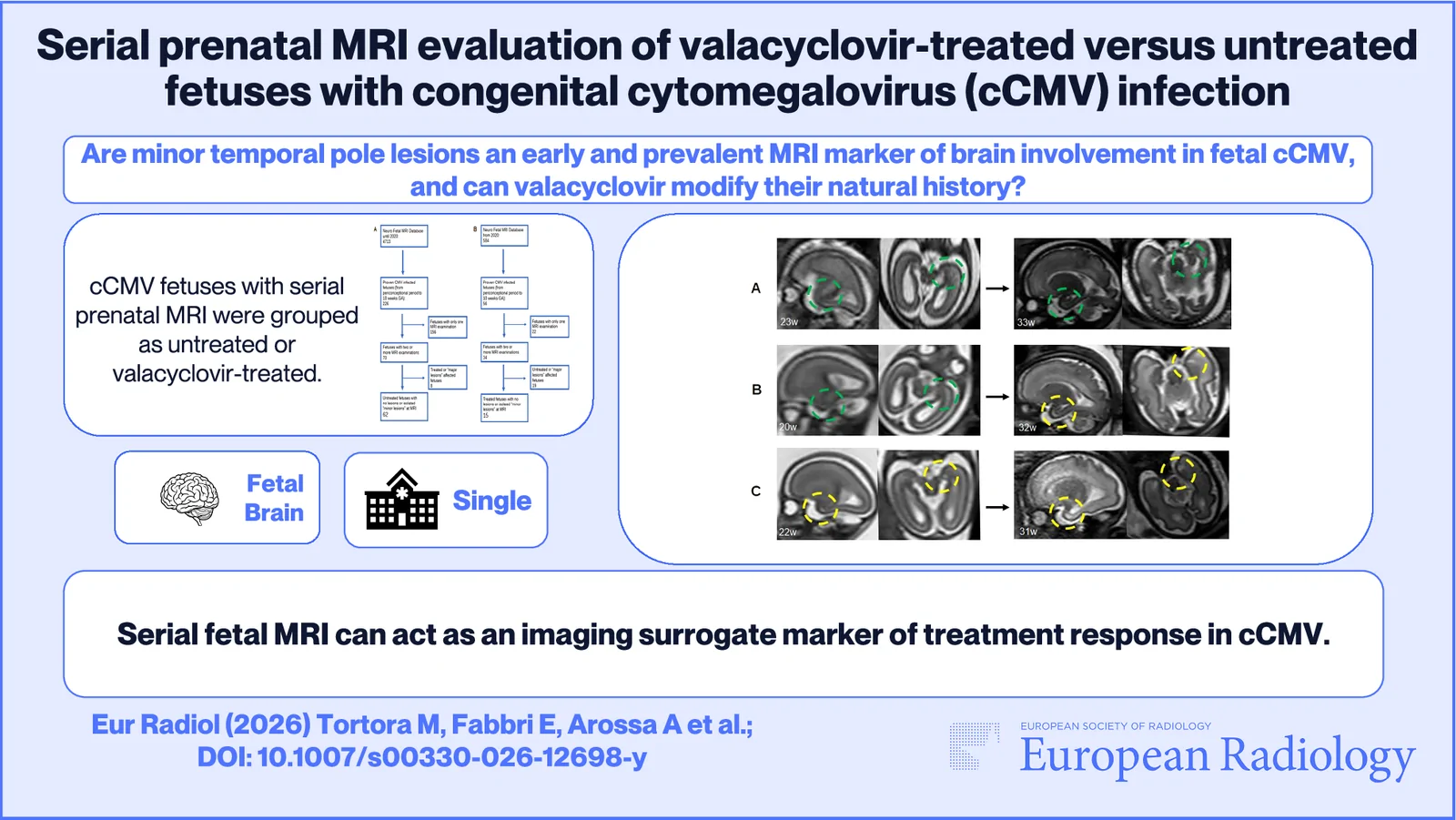

## Introduction

Congenital cytomegalovirus (cCMV) infection remains the leading cause of non-genetic sensorineural hearing loss (SNHL) and neurodevelopmental impairment worldwide. The prevalence of cCMV is approximately 0.5–2% of all live births, with significant geographical variation depending on maternal seroprevalence [1]. While the majority of infected fetuses are asymptomatic at birth, up to 10–15% may develop long-term sequelae, even in the absence of overt clinical signs during the neonatal period [2]. Fetal magnetic resonance imaging (MRI) has become an essential tool in the prenatal evaluation of CMV-infected fetuses [3]. It provides superior contrast resolution compared to ultrasound and can detect subtle brain abnormalities, including isolated “minor” lesions—typically represented by temporal pole T2 hyperintensity and small cystic changes. Although often underestimated, these abnormalities have been associated with subsequent SNHL, unilateral auditory pathway dysfunction, and mild cognitive or behavioral impairment [4].

For the sake of completeness, brain lesions were categorized as “major” or “minor”, defined as follows: “major” lesions include microcephaly, polymicrogyria, cerebellar hypoplasia, severe ventriculomegaly, widespread WM alteration, intracranial calcifications; “minor” findings include polar temporal lesions, subependymal cysts in the ventricular angle region, intraventricular septa, white matter (WM) T2 hyperintensity in regions different from temporal lobes and mild ventriculomegaly (10–12 mm) [4].

Therapeutic strategies aimed at mitigating the impact of CMV infection in utero have been investigated for more than two decades. Oral valacyclovir, a prodrug of acyclovir with increased bioavailability, can achieve higher maternal and fetal plasma concentrations and has been proposed as an antiviral intervention during pregnancy. Previous studies suggested that maternal treatment with high-dose valacyclovir (8 g/day) may reduce viral replication, decrease the risk of severe symptomatic infection at birth, and potentially improve neurological outcome [5, 6]. However, radiological correlates of these effects, especially on subtle “minor” lesions, remain underexplored.

The present study compares serial prenatal MRI findings between valacyclovir-treated and untreated fetuses with confirmed cCMV infection, focusing on the occurrence and progression of isolated “minor” lesions. This focus was intentional, as severe fetal cerebral lesions are known to carry a poor prognosis and are unlikely to be reversible with treatment [7]. Consequently, fetuses with major brain abnormalities were not included in the study population. By specifically selecting cases without severe involvement, our aim was to investigate whether antiviral therapy may influence the development and evolution of subtle MRI abnormalities, which are often underestimated but may still be associated with clinically relevant outcomes. This approach allows for a more appropriate assessment of treatment effects in the subgroup of fetuses in whom therapeutic intervention is most likely to be beneficial.

## Materials and methods

### Study population and design

This was a retrospective single-center cohort study performed on fetuses with confirmed congenital CMV infection who underwent serial brain MRI. All cases were identified from our institutional fetal MRI database. Maternal CMV infection was documented by seroconversion between the periconceptional period and 18 weeks’ gestational age (GA), and fetal infection was confirmed by polymerase chain reaction (PCR) or viral isolation from amniotic fluid. The candidates were recruited as clinical cases with ethical approval for retrospective review of clinical notes and MRI by the Milano Area 1 Ethics Committee (no. 27757/2022). Informed consent to the research and to publication of the results was obtained.

Patients were selected based on predefined criteria. Specifically, inclusion required the availability of at least two serial fetal brain MRI examinations for each case and complete clinical data regarding maternal therapy. Fetuses with severe brain anomalies detected on ultrasound or fetal MRI at the time of the initial assessment were excluded, as treatment was considered unlikely to modify their outcome and in utero therapy is generally recommended only in cases with mild-to-moderate disease [6]. The requirement for serial fetal MRI examinations allowed assessment of the effect of therapy on the appearance and progression of brain lesions compared with the expected natural history of the infection. The timing of the first and second fetal MRI was established a priori and fell within specific GA ranges; therefore, lesions identified at the first examination were classified as early lesions, whereas those detected only at the second examination were defined as late lesions. Neonatal MRI findings and postnatal outcomes were not available for all cases at the time of analysis and will be addressed in a subsequent study.

**Fig. 1:**
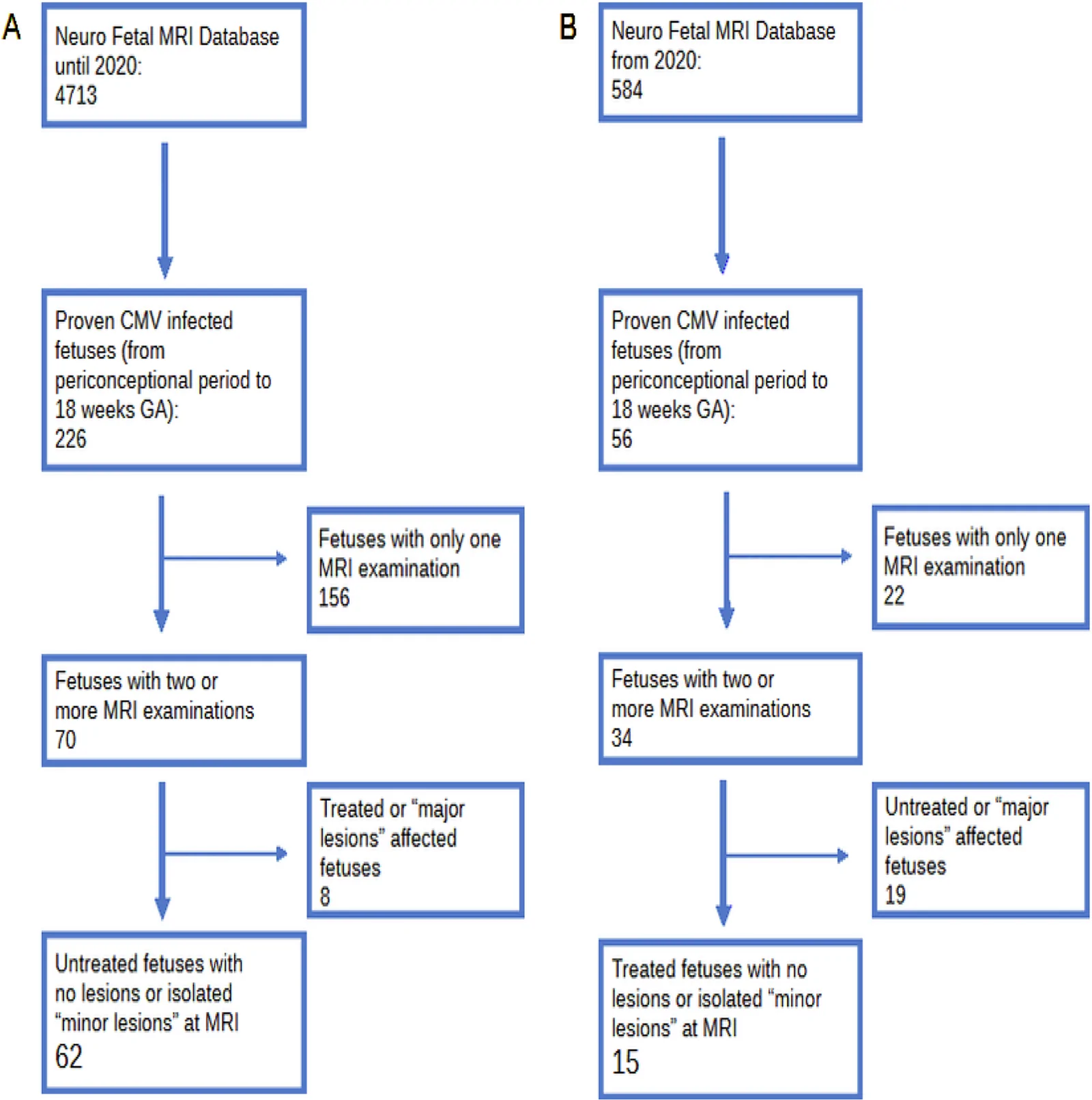
Flowchart illustrating cohort selection. Fetuses with confirmed cCMV infection and at least two prenatal MRI examinations were included and divided into untreated (Group A) and valacyclovir-treated (Group B) cohorts after application of exclusion criteria

In this regard, we have provided a flowchart illustrating the selection of our cohort divided into group A (untreated patients: controls) and group B (treated patients: cases), as shown in Fig. 1.

#### Group A: untreated

From a database of about 4700 fetal MRIs performed between 2005 and 2020, 226 cases were carried out for proven prenatal CMV infection. After exclusion of 156 with only a single MRI, 70 fetuses with at least two MRIs remained. Eight were further excluded because of immunoglobulin treatment or due to the presence of MR major lesions. The final untreated group consisted of 62 fetuses.

#### Group B: treated with valacyclovir

Of the 56 MRIs performed between 2020 and 2024 in pregnancies with confirmed CMV, 22 were excluded due to a single MRI, 12 due to non-adherence to therapy, and 7 due to the presence of major lesions or due to loss at follow-up. The final treated cohort consisted of 15 fetuses with at least two MRIs, confirmed maternal seroconversion, and adherence to oral valacyclovir (8 g/day) from diagnosis until delivery.

This rigorous selection ensured comparability between groups.

### MRI protocol

Examinations were performed as part of the clinical imaging assessment in affected pregnancies, using 1.5-Tesla or 3-T scanners at two different imaging centers with abdominal or cardiac phased-array coils, without maternal-fetal sedation. Standard protocol included multiplanar T2-weighted single-shot sequences (TR/TE 3000/180 ms, in-plane resolution 1.1 × 1.1 mm² for 1.5 T; TR/TE 2000/130 ms, in-plane resolution 0.8 × 0.8 mm² for 3 T; slice thickness 3 mm; field of view 280–320 mm; matrix 256 × 256; number of signal averages 1), T1-weighted fast spin-echo sequences acquired during maternal apnea (TR/TE 600/12 ms; slice thickness 3 mm), steady-state free precession (SSFP/BALANCE) sequences (TR/TE 4/2 ms, flip angle 60°, slice thickness 3 mm, in-plane resolution 1.2 × 1.2 mm²), and diffusion-weighted imaging (DWI) (single-shot echo-planar imaging, *b*-values 0 and 700 s/mm², TR/TE 5000/80 ms, slice thickness 3 mm, matrix 128 × 128). In selected cases, additional single-shot FLAIR sequences were obtained (TR/TE/TI 9000/120/2200 ms, slice thickness 3 mm, matrix 192 × 192). All sequences were acquired in axial, sagittal, and coronal planes, allowing high-resolution assessment of subtle brain abnormalities, including minor lesions such as temporal pole T2 hyperintensity, small cysts, and other subependymal or occipital findings.

### Image review

Two senior neuroradiologists with > 10 years’ experience in fetal neuroimaging reviewed all cases in consensus. The primary outcome measure was the presence of “minor” brain lesions, defined as temporal pole T2 hyperintensity, focal temporal horns dilation, or small cysts without associated major malformations. The evaluations were performed primarily on previously acquired T2-weighted MRI images. Diffusion and T1 images were mentioned as part of the imaging protocol used for global fetal brain evaluation in infected fetuses; however, they were not considered of particular note in our review, as they did not provide additional diagnostic value for the minor lesions under investigation.

Lesion onset was categorized as:

- Absent (both MRIs negative),
- Late (lesions appearing only on 2nd MRI),
- Early (lesions already visible on 1st MRI).

### Statistical analysis

Binary comparisons (absence vs presence of signs) were assessed with Fisher’s exact test. Distribution among three categories (none/late/early) was tested with χ². Odds ratios (OR) and 95% confidence intervals (CI) were calculated. Statistical significance was set at *p* < 0.05.

## Results

### Patient characteristics

Seventy-seven fetuses were included (62 untreated, 15 treated). Baseline maternal and fetal characteristics were comparable between groups (Table 1). All patients started in utero TP before the first MR examination and within the 18th week of gestation, less than six weeks from the presumed date of infection in 8 cases and more than 6 weeks in 7 cases.

**Table 1:** Demographic and baseline characteristics of untreated and valacyclovir-treated fetuses with cCMV infection

Characteristic	Untreated (*n* = 62)	Treated (*n* = 15)	*p* value
Mean maternal age (years)	31.4 ± 4.6	32.1 ± 3.9	0.54
Mean GA at 1st MRI (weeks)	21.5 ± 2.1	21.8 ± 2.0	0.63
Mean GA at 2nd MRI (weeks)	32.6 ± 2.8	32.3 ± 3.0	0.71
Fetal sex (M/F)	34/28	8/7	0.92

Data are reported as mean ± standard deviation or absolute numbers

*GA* gestational age

### MRI findings

All patients belonging to Group A were scanned at 1.5 T either at first or second examination, while among the 30 MRIs performed in total by patients in group B, 20 were performed at 1.5 T and 10 at 3 T, with 4 patients having the exams performed on two different machines (Table 2).

**Table 2:** Distribution of fetal MRI examinations in untreated and valacyclovir-treated groups

MRI	Untreated (*n* = 62)	Treated (*n* = 15)
Total number of MRIs	124	30
MRIs at 1.5 T	124/124	20/30
MRIs at 3 T	0	10/30
Patients with scans on 1.5-T	100%	53.3%
Patients with scans on 1.5-T	0	20.0%
Patients with scans on two machines	0	26.7%

The table shows the total number of MRIs performed, the number performed at 1.5 T and 3 T, and the percentage of patients scanned exclusively on 1.5-T or 3-T scanners, as well as those who underwent examinations on both machines

In the untreated group, 37.1% of fetuses remained MRI-negative, 51.6% developed late-onset minor lesions, and 11.3% showed early-onset lesions. In the treated group, 73.3% remained MRI-negative, 26.7% developed late-onset lesions, and none showed early-onset abnormalities (Table 3).

**Table 3:** Distribution of minor brain lesions on serial prenatal MRI in untreated and valacyclovir-treated fetuses

MRI finding	Untreated (*n* = 62)	Treated (*n* = 15)
Always negative	23 (37.1%)	11 (73.3%)
Lesions only at 2nd MRI (late)	32 (51.6%)	4 (26.7%)
Lesions already at 1st MRI (early)	7 (11.3%)	0 (0%)

Minor lesions included temporal pole T2 hyperintensity, focal temporal horn dilation, and small cysts without associated major malformations

**Fig. 2:**
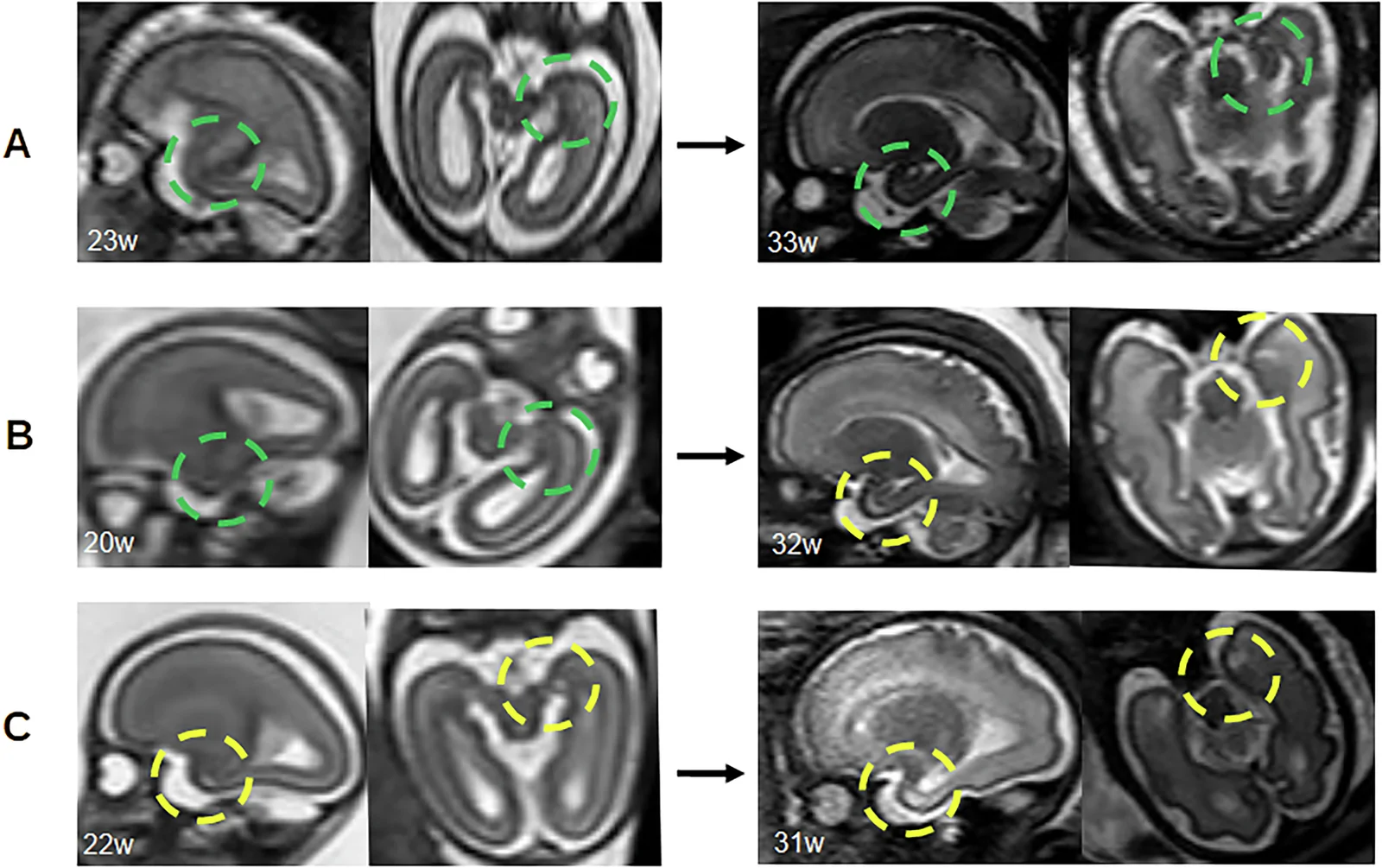
Serial prenatal MRI evolution of CMV-related minor temporal pole lesions in three representative cases. **A** (top row) shows persistently normal MRI findings at 23 and 33 gestational. **B** (middle row) demonstrates late-onset temporal pole T2-weighted hyperintensity appearing on the second prenatal MRI at 32 weeks. **C** (bottom row) shows early-onset temporal pole T2-weighted hyperintensity already visible at 22 weeks, persisting at 31 weeks

Representative cases are shown in Fig. 2.

### Statistical outcomes

A global analysis, considering the three categories (no abnormalities, late positivity, early positivity) and all types of minor lesions (including endoventricular septa and thalamo-caudate sulcus cysts), did not reveal statistically significant differences between the two groups (χ² = 2.54, *p* = 0.28). Similarly, the dichotomous evaluation (never positive vs at least once positive) was not statistically significant: 37.1% of the untreated group remained negative compared to 53.3% of the treated group (χ² = 0.73, *p* = 0.39; Fisher = 0.38). Although not significant, a trend in favor of treatment was observed, with a higher proportion of patients remaining negative and an odds ratio < 1. Finally, the analysis focusing on the time of disease onset (early vs late positivity) showed that 17.9% of untreated patients were already positive at the first MRI, while no early cases were recorded in the treated group. However, this difference also did not reach statistical significance. In summary, in the initial analysis the observed differences—namely, a higher proportion of always-negative patients and the absence of early positivity in the treated group—did not reach statistical significance, likely due to the limited sample size.

When the analysis was restricted to polar temporal lesions (Table 4), different results emerged. Regarding the temporal pole lesions diagnosed in our series, these were always bilateral, with only some signs of asymmetry represented by a possible different degree of dilation of the temporal horns or by the presence of cystic aspects on one side and simple dilation of the temporal horn on the other.

**Table 4:** Summary of statistical analyses comparing MRI findings between untreated and valacyclovir-treated fetuses

Analysis	Untreated	Treated	Test	*p* value	OR (95% CI)
Always negative vs any lesions	23/62	11/15	Fisher	0.018	4.67 (1.29–16.9)
3-Category distribution (none/late/early)	–	–	χ²	0.02	–

Statistical significance was set at *p* < 0.05

*OR* odds ratio, *CI* confidence interval

We also observed subependymal cysts in the ventricular angle region in two cases, and endoventricular occipital septa in one case.

The binary comparison between patients who developed abnormalities and those who did not showed a significant difference: 73.3% of treated patients remained negative compared with 37.1% of untreated patients (Fisher *p* < 0.05), with an odds ratio of 4.67 (95% CI: 1.29–16.9). This indicates that treated patients had an approximately fivefold higher likelihood of remaining free of radiological abnormalities compared to untreated patients. The more granular three-category analysis also confirmed statistical significance (χ², *p* = 0.02): treated patients showed fewer progressions and no early abnormalities. In conclusion, while the preliminary analyses did not reach statistical significance, the focused approach on polar temporal lesions highlighted a clear benefit of treatment, with a significantly higher probability of remaining free of infection signs and of the absence of early positivity.

## Discussion

### Principal findings

This study provides evidence that maternal valacyclovir therapy is associated with a higher proportion of fetuses remaining MRI-negative during pregnancy, and with the absence of early “minor” brain lesions. The protective effect was statistically significant, despite the relatively small number of treated cases, suggesting a robust signal.

### Review of the literature on valacyclovir therapy

#### Clinical outcomes

The first randomized controlled trial by Leruez-Ville and colleagues [6] demonstrated that maternal valacyclovir significantly reduced the risk of fetal infection when administered early after maternal primary infection. Faure-Bardon et al [8] extended these findings, reporting a higher proportion of asymptomatic neonates and reduced viral load in treated pregnancies.

More recently, observational studies and meta-analyses have confirmed a modest but consistent benefit in terms of reducing symptomatic disease at birth and improving perinatal outcomes. However, most of these studies focused on clinical endpoints such as hepatosplenomegaly, intrauterine growth restriction, or hearing loss, rather than neuroimaging correlates [9, 10].

#### Radiological outcomes

Few studies have directly addressed the impact of therapy on fetal brain imaging. In small cohorts, valacyclovir was associated with reduced progression of ventricular dilation and delayed onset of parenchymal abnormalities, although statistical significance was rarely reached [11, 12]. Our study adds to this limited body of evidence by systematically analyzing serial MRI findings with a focus on subtle lesions.

### The prognostic role of “minor” temporal pole lesions

Temporal pole T2 hyperintensity is increasingly recognized as the most frequent subtle imaging feature of cCMV. Although once considered benign, accumulating evidence—including our previous work—indicates that these lesions may predict unilateral SNHL or mild cognitive impairment. In that study of 36 fetuses, the frequency of minor lesions increased with GA, and 57% of children developed mild/moderate sequelae at long-term follow-up, including 34% with unilateral hearing loss [4]. Thus, reducing or preventing the occurrence of temporal pole lesions should not be regarded merely as a radiological achievement, but rather as a possible indicator of a milder overall cerebral involvement. This condition may, in turn, translate into a lower risk of long-term neurodevelopmental sequelae, including auditory dysfunction. Although human cytomegalovirus (CMV) demonstrates a well-documented tropism for the temporal lobes, hippocampus, and auditory pathways [13–15], current evidence suggests that CMV-related hearing loss primarily results from direct viral and immune-mediated injury to the cochlear sensory epithelium and spiral ganglion neurons, rather than from cortical or subcortical lesions [16–18]. Therefore, the observed association between minor brain abnormalities and hearing impairment is more likely to reflect the overall burden of congenital infection than a direct causal effect of temporal lobe involvement.

### How therapy may influence lesion development

The beneficial effect of valacyclovir on the development of brain lesions in fetuses with congenital CMV infection is probably mediated through multiple, partially overlapping mechanisms [19]. First, by inhibiting viral replication, valacyclovir reduces the overall viral burden both at the placental level and in the fetus. This decreased viral load limits the amount of virus that can reach the developing brain, thereby reducing the probability and extent of direct viral injury to neural tissue [6].

Second, suppression of viral activity may influence the timing of lesion appearance. By delaying or attenuating viral aggression during critical periods of brain maturation—such as neuronal migration and cortical organization—the drug may postpone the onset of parenchymal damage beyond the most vulnerable developmental windows. This temporal shift could translate into a substantial reduction in the severity of long-term neurological sequelae [5].

Third, therapy may act indirectly by modulating the host immune response. CMV-related injury is not exclusively the result of direct viral cytopathic effects but is also amplified by secondary inflammatory cascades. By lowering viral replication, valacyclovir likely decreases the magnitude of the maternal and fetal inflammatory response, thereby limiting immune-mediated tissue damage. This effect may be particularly relevant in regions that appear highly susceptible to inflammation, such as the temporal poles, where focal lesions are often observed [20].

Taken together, these mechanisms suggest that the impact of valacyclovir is not limited to simple viral suppression but extends to the modulation of both the timing and the pathophysiological processes underlying lesion formation, with potential long-term benefits for neurodevelopmental outcomes.

### Broader implications: linking minor lesions and hearing outcomes

SNHL is the most frequent long-term consequence of cCMV. Even in children born asymptomatic, the risk of delayed-onset SNHL remains significant (up to 15%) [21]. Minor MRI lesions, especially temporal pole abnormalities, may serve as early surrogate markers for auditory involvement [4]. Preventing these lesions through therapy could therefore reduce not only radiological abnormalities but also the incidence of hearing loss.

Indeed, studies correlating MRI findings with audiological outcomes have shown that children with minor polar lesions are more likely to present unilateral hearing loss during follow-up. Our findings of absent early lesions in the treated group provide encouraging indirect evidence that valacyclovir may also protect auditory structures. Further studies considering auditory outcomes in valacyclovir-treated patients are needed to confirm this hypothesis.

### Clinical and counseling perspectives

From a clinical standpoint, the results support the integration of MRI into prenatal monitoring of treated pregnancies. Serial imaging allows detection of late-onset lesions, refining counseling for parents. The finding that 73% of treated fetuses remained lesion-free emphasizes the potential for improved prognostic reassurance [22, 23]. Furthermore, highlighting the absence of early lesions may be critical during prenatal counseling, as early positivity often indicates a higher risk for neurodevelopmental sequelae.

### Limitations

The main limitation is the relatively small number of treated cases, reflecting the recent introduction of valacyclovir into clinical practice and the strict inclusion criteria. Retrospective design and heterogeneity of imaging platforms represent additional constraints. Nevertheless, the statistically significant findings despite limited numbers strengthen the clinical relevance of the observed trends.

Another limitation of the present study is that, within the group of treated patients, the fetal MRI examinations were performed using different scanners (1.5 T vs 3 T) and at two separate imaging centers. Although standardized protocols were applied, differences in field strength, coil type, and scanner performance may have affected image quality and the detection of subtle brain abnormalities. This variability represents an additional limitation, as it could have influenced the assessment of minor lesions and their apparent progression over time. Furthermore, the difference in the range of MRI acquisition dates between the treated and untreated groups, linked to the more recent availability of in utero therapy, also represents a limitation, as it may introduce potential temporal bias in imaging and case selection.

### Future directions

Future research should focus on multicenter, prospective trials to further clarify the role of valacyclovir in congenital CMV infection. Such studies are needed to confirm the protective effect of the therapy on MRI outcomes, to better understand the relationship between dosage and treatment duration, and to determine the optimal therapeutic regimen.

Additionally, it will be important to correlate imaging findings with long-term clinical outcomes, including both audiological and neurocognitive development, in order to establish the clinical relevance of observed brain changes. Finally, identifying reliable biomarkers—such as viral load measurements or immunological markers—may help predict the evolution of brain lesions and guide individualized treatment strategies.

## Conclusion

Prenatal valacyclovir therapy in fetuses with confirmed CMV infection was associated with a significantly higher rate of persistently negative MRI findings and complete absence of early-onset minor brain lesions. Given the known association between temporal pole abnormalities and subsequent SNHL and cognitive impairment, these findings suggest that maternal therapy may not only modify radiological trajectories but also reduce the risk of long-term auditory and neurological sequelae. Larger, prospective studies are warranted to validate these results and establish imaging as a surrogate marker of therapeutic efficacy.

## Abbreviations

cCMV: Congenital cytomegalovirus
CMV: Cytomegalovirus
GA: Gestational age
MRI: Magnetic resonance imaging
SNHL: Sensorineural hearing loss
